# Mannose supplementation in PMM2-CDG

**DOI:** 10.1186/s13023-021-01988-x

**Published:** 2021-08-11

**Authors:** Roman Taday, Julien H. Park, Marianne Grüneberg, Ingrid DuChesne, Janine Reunert, Thorsten Marquardt

**Affiliations:** Department of General Pediatrics, University Children’s Hospital Münster, Albert-Schweitzer-Campus 1, 48149 Münster, Germany

**Keywords:** PMM2, Congenital disorder of glycosylation, Mannose, Galactose, Therapy

## Abstract

In this response to the letter by Witters et al., we refer to the authors' arguments regarding spontaneous enhancement of glycosylation and the claim, that mannose has no place in the treatment of PMM2-CDG. Our paper “Dietary mannose supplementation in phosphomannomutase 2 deficiency (PMM2-CDG)” has shown that further investigation of mannose in PMM2-CDG is worthwhile alongside other treatment options and should not be dismissed off-hand without the willingness to prove or disprove it in controlled prospective clinical trials.

The letter by Witters et al. relates to our recent publication on long-term mannose supplementation, a paper that did not summarize data of a prospective clinical study but analyzed data obtained by long-term care for PMM2 patients [1, 2]. Many of their arguments had already been discussed in our paper and our response is lined out in the following addresses the critical points.

## Improvement of glycosylation and biochemical variables

The letter by Witters et al. regarding our publication focuses on the fact that glycosylation in PMM2-patients may improve spontaneoulsy over time. This is well known, not disputed, and has been cited throughout our study.

In their letter, the authors present the development of transferrin glycosylation in a cohort of 37 patients with somewhat limited data of 3 individual measurements per patient in average. In contrast, the subjects of our study underwent transferrin glycosylation analysis up to 40 times during the follow-up period. The use of the di- and monoglycosylated transferrin ratio limits the comparability of the studies since it does not take aglycosylated (i.e. asialo-) transferrin into account.

Two observations are critical in the interpretation of the data we have presented but have not been addressed by Witters et al.:

1. Following long-term interruption of mannose supplementation, transferrin glycosylation returned to pre-treatment levels as demonstrated in Fig. 1C of the original publication. This was mirrored clinically by nerve conduction velocities improving during supplementation and decreasing again following discontinuation.
2. The observed improvement of glycosylation was age-independent.

## Clinical improvement

In our paper, we state that due to the highly variable clinical manifestations and course as well as laboratory abnormalities, it is not easy to make predictions about the outcome or natural history of an individual patient a priori or to define relevant end points for clinical trials. Many arguments raised in the letter do not respect the fact that our study summarizes historical data from clinical practice and not from a prospective study. Regarding the deterioration of normalized nerve conduction velocity of our index-patient under mannose, Witters et al. do not discuss that deterioration happened in a temporal relation to discontinuation of mannose supplementation at the age of nine years. We equally conclude double-blind randomized controlled trials taking into account the natural history of the disease will be needed to validate the current findings.

### Role of mannose in PMM2-CDG

Given the efficacy of mannose supplementation in cell culture [4, 5] as well as in a murine model [6] in addition to our clinical findings, we are very concerned about the implied idea that studying mannose in PMM2-CDG might be a futile endeavor and that other treatment options might be the only ones worth pursuing in the future. Dismissing ideas off-hand without the willingness to prove or disprove them in a clinical trial, is not what clinical science should be like. As the authors state themselves, there are more than 900 patients worldwide providing a basis for many different treatment trials. Indeed, even non-intuitive concepts such as D-galactose supplementation for PMM2-CDG, a disorder in which no hypogalactosylation has ever been found, have been explored [3].

Alternative therapeutic concepts put forward by the authors are hindered by their own limitations: while showing impressive improvement with regards to ataxia, acetazolamide is a purely symptomatic therapy targeting a single one of the multiple organ systems involved in PMM2-CDG [7]. Mannose derivates such as Man-1-P are in theory intriguing since they might bypass the enzyme defect. In reality, there are currently no stable, non-toxic, and easily transportable compounds for lifelong therapy [8] in addition to the limitation that these compounds are likely not able to cross the blood–brain barrier (BBB).

In contrast, small molecule PMM2 activators such as chaperones are in principle able to pass the BBB but are currently limited to specific mutations, [9] as demonstrated by the aldolase inhibitor epalrestat [10]. MPI-inhibitors have limited access to some tissue compartments or are effective only in areas with a favorable PMM2: MPI-ratio [8].

Mannose ensures a half-life suitable for oral continuous therapy, as well as an all-encompassing accessibility to different organ systems (including the blood–brain barrier) [11]. A goal of therapy for PMM2-deficient patients might be to increase the flux of metabolic precursors into the depleted glycosylation pathways. Combinations of dietary mannose with other mentioned coadministratives may be viable options for establishing appropriate continuous therapy. Our paper gives sufficient evidence that mannose could be a cornerstone for clinically applicable treatment options for PMM2-CDG. Nevertheless, especially in these times, where truth is often difficult to determine, no treatment idea should be dismissed off-hand but all should be proven or disproven by well-designed clinical trials.

The vivid discussion around potential therapies stresses the desperate need for improved treatment options for PMM2-CDG and indeed most glycosylation disorders. The ongoing efforts of multiple groups to advance the treatment for affected individuals are encouraging and offer the potential to impact patient care substantially, as does the impressive involvement of patient organizations. We agree that natural history studies and novel biomarkers will be key to design the urgently needed trials to validate therapeutic concepts for CDG and would like to stress the importance to consider all available options to improve treatment of these devastating diseases.

## Data Availability

Not applicable.
